# The relationship between school bullying victimization and social mindfulness in middle school students: a chain mediating model of self-concept clarity and cognition reappraisal

**DOI:** 10.3389/fpsyg.2024.1388301

**Published:** 2024-08-05

**Authors:** Weijing Yang, Dong Hu, Ying Guo

**Affiliations:** School of Psychology, Sichuan Normal University, Chengdu, China

**Keywords:** bullying victimization, social mindfulness, self-concept clarity, cognition reappraisal, adolescence

## Abstract

**Objective:**

To determine the relationship between school bullying victimization and social mindfulness and its mechanism in light of the interdependence and schema theories.

**Method:**

The Chinese version of the Delaware Bullying Victimization Scale-student, Emotion Regulation Questionnaire, Self-Concept Clarity Scale and The Social Mindfulness Self-report Scale (SMSRS) were distributed to 553 middle school students.

**Results:**

(1) The correlations of school bullying victimization with social mindfulness, self-concept clarity, and cognition reappraisal were statistically significant. (2) School bullying victimization had a significant effect on social mindfulness. (3) The simple mediating role of self-concept clarity and cognition reappraisal between school bullying victimization and social mindfulness were significant. (4) Self-concept clarity and cognition reappraisal played a chain mediating role between school bullying victimization and social mindfulness.

## 1 Introduction

Persons who are willing to express kindness, respect, and concern for others’ interests and needs and even leave room for others to choose what they like in interpersonal interactions are assumed to have a high level of social mindfulness (Van Lange and Van Doesum, 2015). The term, social mindfulness was introduced in a Dutch study (Van Doesum et al., 2013). It is measured by the SoMi paradigm, as per which a person with a high level of social mindfulness chooses one object from the same two rather than the unique one (Van Doesum et al., 2013). It is a kind of prosocial behavior with a low cost that coordinates with prosocial value orientation and is highly recommended all over the world (Van Doesum et al., 2021). In China, social mindfulness is both an interpersonal trait reflecting Chinese culture and a mental skill and motivation expressing kindness in interpersonal interactions (Tian et al., 2021). It is part of the Chinese core valuation. It not only means that we should be kind and respect others while doing good but also represents equality, openness, optimism, and enterprising (Tian et al., 2021). Previous studies indicated that social mindfulness promotes cooperation (Dou et al., 2018). People with high social mindfulness are easy to get along with and tend to be other-regarding (Van Doesum et al., 2013). Social mindfulness results in positive emotion, enhanced well-being, and repair of damaged trust in the recipients (Nie, 2018). In sum it is the expression of altruism (Dou et al., 2017). Prosocial tendencies tend to develop in the period of adolescence (Yoo et al., 2013). At this stage, young people want to be kind and honest so as to be appreciated by others (Damon and Hart, 1992). That is to say they would like to be a person with social mindfulness. The development of social mindfulness depends on how adolescents interact with their friends and corresponding responses (Yan et al., 2022). However, the victims of bullying incidents normally have a low acceptance rate and poor peer relationships (Perren and Hornung, 2005) and have a lower tendency to express kind and help (Coulombe, 2021). Does that mean bullied experience would affect the development of their social mindfulness? If so, what’s the core mechanism? This study is trying to explore the relationship between school bullying victimization and social mindfulness and its mechanism.

Bullying at school occurs when some students are attacked intentionally by the perpetrators (Xie et al., 2018). The interdependence theory indicates that the quality of social interactions is influenced by the behavioral outcomes of both parties involved (Rusbult and Van Lange, 2003). If the behavior of a person has a positive impact on another person, the latter will also engage in appropriate behaviors to meet the needs of the former. The inner desires of adolescents are satisfied with social support and companionship, but bullying victims are deprived of these aspects, leading to the slow development of their social mindfulness (Wo et al., 2001). The theory of mind, the basic ability of social mindfulness, lags because of heartbreaking experiences (Renouf et al., 2010; Yan et al., 2022), and adolescents who face rejection rarely tend to behave altruistically. They normally show insensitivity to physical and emotional pain of others, have low desire to donate, and are hardly willing to share knowledge (DeWall and Baumeister, 2006; Kothgassner et al., 2017; Howard et al., 2020; Takhsha et al., 2020). Therefore, it is logical to predict a negative correlation between bullying victimization and social mindfulness.

According to the schema therapy model, children develop different emotional needs due to variations in experiences and temperament during their early childhood. If these needs are not met, they may develop maladaptive early attachment patterns, including self-defeating cognitive styles and underdeveloped self-concepts (Young et al., 2003). Self-concept clarity, positively related to one’s self-esteem, is defined by how clearly and confidently one knows oneself as well as the internal consistency and temporal stability of one’s core self-concept (Campbell et al., 1996). In his book ‘Identity: Youth and Crisis,’ Erikson mentioned that the core responsibility of adolescence is to develop a clear sense of self, meaning adolescents are trying to figure out who they are and the life goals they are determined to achieve. Previous studies indicate that children with clear self-concept are empathetic, willing to put themselves in others’ shoes and would like to make proactive efforts to cultivate healthy interpersonal relationships (Beyers and Seiffge-Krenke, 2010; Kural and Kovács, 2022). Teenagers who have a consistent and clear view of themselves report lower levels of anxiety and depression and better interpersonal relationships (Van Dijk et al., 2014; Becht et al., 2017) than their counterparts who do not. Most importantly, a strong, stable, and clear sense of self may allow adolescents to have more empathy toward others in distress, leading to more helpful behaviors (Krol and Bartz, 2022). However, those being bullied normally acquire lower peer acceptance rate, leading to a lower self-evaluation value and self-injury tendency (van Geel et al., 2015). Negative experiences in the past can also induce cognitive vulnerability, triggering unhealthy cognitive patterns and leading to doubts about one’s self-identity and sense of self (Geng et al., 2022). Further, previous studies have reported that childhood trauma, negative life experience, and rejection are predictors of unclear self-concept later in life (Ayduk et al., 2009; Evans et al., 2015; Wong et al., 2019). Thus, we suggest that the link between school bullying victimization and social mindfulness is mediated by self-concept clarity. Specifically, school bullying victimization is related to a low level of self-concept clarity, which in turn is related to low social mindfulness.

Cognitive reappraisal is a positive emotional regulatory strategy whereby an individual alters the trajectory of an emotional response through a reinterpretation of the meaning of surrounding stimulus (Gross, 1998); it gradually matures across adolescence (Silvers et al., 2012). According to the schema theory, children who experience bullying may struggle to use adaptive emotion regulation strategies such as cognitive reappraisal to positively evaluate the significance of events, leading to a decrease in their ability to experience joy (Young et al., 2003). For example, Gardner et al. (2017) suggest that poor cognitive reappraisal of emotions is associated with a high possibility of using maladaptive coping strategies; in turn, this relationship mediates the relationships between peer victimization and school loneliness. Völlink et al. (2013) state that victims of cyberbullying have the highest score on depressive coping and tend to remain vulnerable to attack and abuse. In the long term, this results in a vicious cycle whereby bullies continue to bully and victims continue to cope negatively and ineffectively. In addition, related studies have shown that adolescents with childhood maltreatment and trauma experiences barely use cognitive reappraisal strategies (Zhang et al., 2005; Yu et al., 2022). Although experiencing bullying can weaken a person’s ability to use cognitive reappraisal strategies, employing these can indeed help adolescents reduce negative emotions and problematic behaviors (Zhan et al., 2017; Chen et al., 2020). For example, positive emotion regulation strategies are vital for reducing the experience of negative affect, and individuals with such strategies have been found to show high levels of prosocial behaviors (Hein et al., 2018). Moreover, a meta-analysis revealed that reappraisal and empathic perspective-taking are highly correlated and both of them rely on shared neural networks (Morawetz et al., 2022), while a high level of perspective-taking indicates a high level of social mindfulness. Thus, we can suggest that school bullying experience hinders victims’ ability to employ cognition reappraisal, in turn influencing the development of their social mindfulness.

Additionally, high self-concept clarity facilitates positive reappraisal because it allows one to engage and process self-relevant information whereas low self-concept clarity hinders emotion regulation strategies through disengagement (Isidro, 2021). Previous research revealed that teenagers with higher self-concept clarity were able to manage negative emotional states properly in response to stressful situations (Parise et al., 2019). Baumgardner (1990) also suggested that individuals with higher self-concept clarity may have more reaction options as they are more aware of their strength and weakness and consequently be more equipped to correctly respond to the demands of negative situations. Combined with the above studies, it can be hypothesized that the school bullying victimization experience influences social mindfulness by affecting an individual’s self-concept clarity and cognition reappraisal skills.

To summarize, this study constructed a chain mediating model to clarify the impact of school bullying victimization on social mindfulness. Based on existing theoretical and empirical studies, this study hypothesized that (1) school bullying victimization significantly and negatively predicts social mindfulness, (2) self-concept clarity mediates the relationship between school bullying victimization and social mindfulness, (3) cognition reappraisal mediates the relationship between school bullying victimization and social mindfulness, and (4) self-concept clarity and cognition reappraisal play a chain mediating role between school bullying victimization and social mindfulness.

## 2 Materials and methods

### 2.1 Participants

Bullying incidents increases in Chinses middle schools (Zhang and Jiang, 2022). Approximately half of all bullying incidents occurred in middle school (Wang et al., 2017; Yang et al., 2017). Thus, we recruited the middle school students as our research participants.

This current study adopted the convenience sampling method. Before distributing questionnaires, we randomly selected two local middle schools, Sichuan Province, China. After obtaining the approval of school principals, we recruited students from grades 7 and 8 on the investigation day. Students in Grade 9 were not recruited due to their busy academic schedules. This study received approval from the Ethic Committee of the college of Psychology of Sichuan Normal University. And written informed consent and approval in this study was provided by the participants and their legal guardians. A total of 600 questionnaires were distributed and 47 were excluded, of which 22 questionnaires had the same option selected more than 10 times in a row and 25 questionnaires had at least three questions omitted. The final analysis included 553 valid questionnaires, yielding an effective response rate of 92.17%. The mean age of all students was 13.87 ± 1.44 years, with 263 (47.56%) boys and 290 (52.44%) girls.

### 2.2 Tools

#### 2.2.1 Bullying victimization

The Chinese version of the Delaware Bullying Victimization Scale-student (DBVS-S) was used to measure the frequency of bullying victimization. This scale was translated and revised by Xie et al. (2018). It comprises 17 items; the 13th item, “I was bullied at this school,” was not included in the data analysis process as it was used as a screening item. Participants are expected to respond to each item on a Likert 6-point scale, 0 = never, 1 = sometimes, 2 = once/twice a month, 3 = once a week, 4 = many times a week and 5 = every day, with higher scores indicating a higher level of bullying victimization. The Cronbach’s alpha for this scale in this study was 0.90.

#### 2.2.2 Self-concept clarity

Self-Concept Clarity Scale (SCC) was developed by Campbell et al. (1996) and revised by Chen and Ouyang (2013). It consists of 12 items (1 = strongly disagree, 5 = strongly agree) and measures participants self-concept clarity. All items are reverse scored except the 6th and the 11th. A higher score indicates a higher level of self-concept clarity. The Cronbach’s alpha for this scale in this study was 0.82.

#### 2.2.3 Cognition reappraisal

We used the cognitive reappraisal scale revised by Chen et al. (2020) for adolescents from the original Emotion Regulation Questionnaire developed by Gross and John (2003). The scale consists of two subscales: cognitive reappraisal and expression inhibition. The cognitive reappraisal rating scale includes six items, and the scores range from 1 (“completely disagree”) to 7 (“completely agree”). Higher scores indicate a higher frequency of the use of cognition reappraisal strategies. The Cronbach’s alpha for this scale in this study was 0.89.

#### 2.2.4 Social mindfulness

Social Mindfulness Self-Report Scale has a two-order structure, including four factors: kindness and respect, inclusive and understanding, positive and open, and humility (Tian et al., 2021; Guo and You, 2024). It consists of 17 items. As all participants are middle school students, some items, such as “I am optimistic and cheerful about my life and work,” were modified to “I am optimistic and cheerful about my life and schoolwork.” The participants were then asked to rate the degree to which these statements fit their daily psychology or behaviors (1 = very badly, 5 = very well). Higher score represents a higher level of social mindfulness. The Cronbach’s alpha for this scale in this study was 0.93.

### 2.3 Statistical analysis

The study conducted the statistical analysis using SPSS version 25.0. Initially, Harman’s single-factor analysis was used to check for common method analysis. Then, descriptive analysis and Pearson correlation analysis were performed to estimate the means, standardized deviations, and correlations among school bullying victimization, self-concept clarity, cognitive reappraisal and social mindfulness. Finally, based on the results of the correlation analysis and our hypotheses, a linear regression model was developed to test the relationship between school bullying victimization and social mindfulness in middle school students. To examine the serial multiple mediation effects of self-concept clarity and cognitive reappraisal between school bullying victimization and social mindfulness, we adopted the SPSS PROCESS macro program (Model 6) designed by Hayes (2017) to complete data analyses. A *p*-value of 0.05 was considered statistically significant. The mediation bootstrapping analysis was conducted using 5,000 resamples and we set bootstrap confidence interval (CI) at 95%.

## 3 Results

### 3.1 Common method biases test

As the data collected relied on self-reports of the participants, covariates might exist. Harman’s one-factor test was used to test the common method bias. The results of the unrotated exploratory factor analysis extracted a total of 10 factors with eigenvalues greater than one, and the first factor explained a 22.65% variation, which is significantly less than the empirical criterion of 40% (Podsakoff et al., 2003), confirming that the result was free from common method bias.

### 3.2 Descriptive statistics and correlational analysis

Table 1 presents descriptive statistics and results of Pearson’s correlation analysis for each variable. The results showed that school bullying victimization was significantly negatively correlated with self-concept clarity (*r* = −0.26, *p* < 0.01), cognitive reappraisal (*r* = −0.16, *p* < 0.01), and social mindfulness (*r* = −0.22, *p* < 0.01); self-concept clarity was significantly positively related with cognitive reappraisal (*r* = 0.23, *p* < 0.01) and social mindfulness (*r* = 0.33, *p* < 0.01); and cognitive reappraisal was significantly positively correlated with social mindfulness (*r* = 0.59, *p* < 0.01).

**Table 1:** Means, standard deviations, and correlation coefficient.

	1	2	3	4	5	6
1. Sex	–					
2. Age	0.42**	–				
3. School Bullying Victimization	−0.11*	−0.07	–			
4. Self-Concept Clarity	0.04	−0.09^*^	−0.26**	–		
5. Cognition Reappraisal	0.03	−0.02	−0.16**	0.23**	–	
6. Social Mindfulness	0.09*	0.02	−0.22**	0.33**	0.59**	–
*M*	1.52	13.87	21.17	36.41	30.79	65.13
*SD*	0.50	1.44	8.05	8.40	6.67	10.59

M, mean; SD, standard deviation; *p < 0.05, **p < 0.01, ***p < 0.001.

### 3.3 Mediation effect test

Based on the correlation analysis, the multiple mediating effect of self-concept clarity and cognitive reappraisal between school bullying victimization and social mindfulness was examined using the SPSS macro-Model 6 after controlling for the demographic variables of sex and age. To test the mediating role of self-concept clarity and cognition reappraisal in the relationship between school bullying victimization and social mindfulness, three equations were used. As shown in Table 2, school bullying victimization had a directly and negatively significant impact on the level of adolescents’ social mindfulness (*β* = −0.10, *p* < 0.05) in equation 3, self-concept clarity (*β* = −0.27, *p* < 0.001) in equation 1, and cognitive reappraisal (*β* = −0.09, *p* < 0.05) in equation 2. Furthermore, the SCC significantly and directly predicted cognitive reappraisal (*β* = 0.16, *p* < 0.001) in equation 2. Finally, SCC (*β* = 0.24, *p* < 0.001) and cognitive reappraisal (*β* = 0.84, *p* < 0.001) could predict social mindfulness positively and significantly in equation 3.

**Table 2:** Regression analysis of variable relationships.

Predictive variable	Model1: self-concept clarity		Model 2: cognition reappraisal		Model 3: social mindfulness	
	*β*	*t*	*β*	*t*	*β*	*t*
Sex	1.15	1.52	0.30	0.49	0.98	1.26
Age	−0.81	−3.08^**^	−0.09	−0.44	0.19	0.71
School bullying victimization	−0.27	−6.29 ^***^	−0.09	−2.56^*^	−0.10	−2.20^*^
Self-concept clarity			0.16	4.52 ^***^	0.24	5.46^***^
Cognitive reappraisal					0.84	15.45^***^
*R^2^*	0.08		0.06		0.40	
*F*	16.35		9.23		71.89	

*p < 0.05, **p < 0.01, ***p < 0.001.

**Figure 1:**
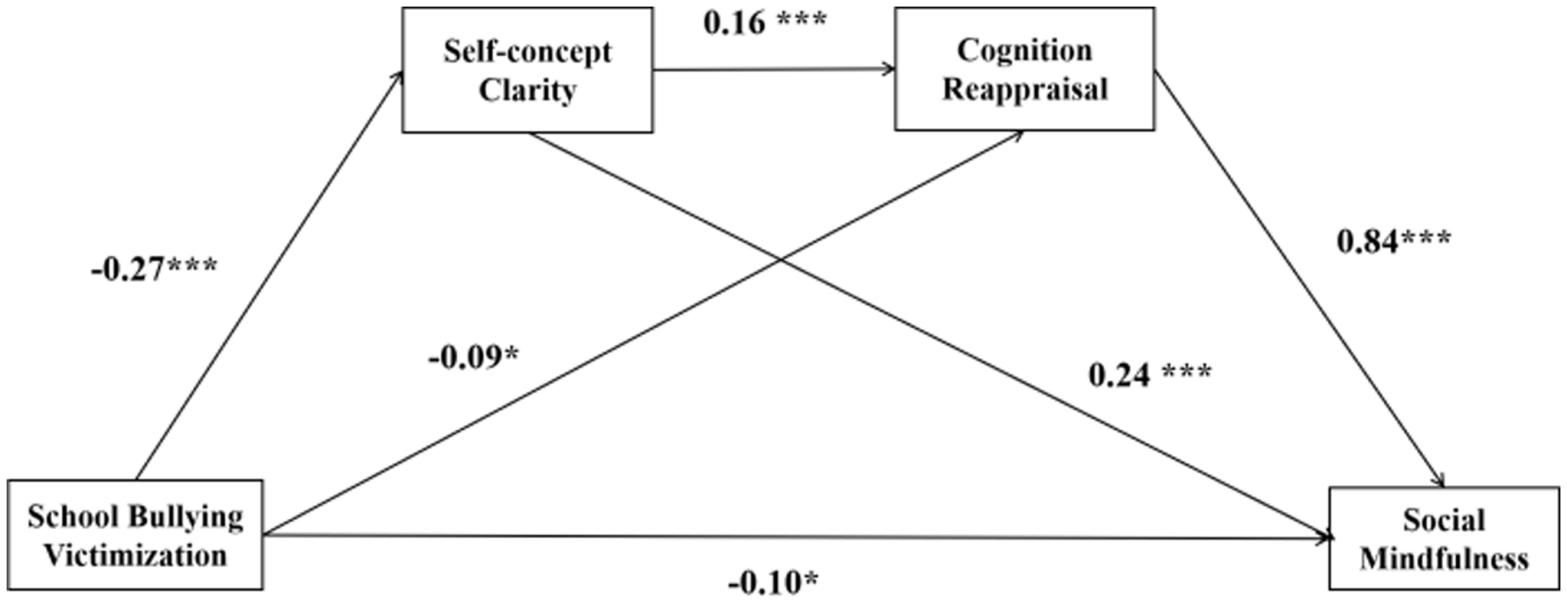
Chain mediation model with unstandardized coefficient. *p < 0.05, **p < 0.01, ***p < 0.001.

Table 3 and Figure 1 show the results of the chain mediating effect of SCC and cognitive appraisal. The 95% CIs of all three indirect effects did not include zero, confirming the significant indirect effects. The total indirect effect was −0.18, accounting for 64.29% of the total effect, and − 0.28 in the relationship between school bullying victimization and social mindfulness. Thus, SCC and cognition reappraisal play a role in mediating the chain effect of school bullying victimization on the social mindfulness of middle school students.

**Table 3:** The effects after bootstrapping from school bullying victimization to social mindfulness.

Path	Effect value	*BootSE*	*BootLLCI*	*BootULCI*	Proportion of relative effect
1. SBV → SCC → SM	−0.07	0.02	−0.1028	−0.0359	25.00%
2. SBV → CR → SM	−0.08	0.04	−0.1513	−0.0026	28.57%
3. SBV → SCC → CR → SM	−0.03	0.01	−0.0611	−0.0165	10.72%
4. Total indirect effect	−0.18	0.04	−0.2626	−0.099	64.29%
5. Direct effect	−0.10	0.05	−0.1908	−0.0109	35.71%
6. Total effect	−0.28	0.06	−0.3864	−0.1705	100%

SBV, school bullying victimization; SCC, self-concept clarity; CR, cognition reappraisal; SM, social mindfulness.

## 4 Discussion

We conducted a cross-sectional study to explore the relationship of school bulling victimization with SCC, cognition reappraisal, and social mindfulness and analyze the chain mediating role of SCC and cognition reappraisal.

### 4.1 The influence of school bullying victimization on social mindfulness

The results revealed that bullying victimization experience was negatively correlated with social mindfulness, which means, victims tend to show lower level of social mindfulness. The interdependence theory suggests that those being bullied lack the opportunities to improve their interaction skills because they receive verbal abuse and ostracism rather than companionship and support from their peers at school; therefore, they do not interact with others in a kind manner (Rusbult and Van Lange, 2003). On the one hand, a safe and positive school environment provides a foundation for adolescents’ prosocial and moral development. However, experiencing bullying can make individuals feel that their safety is not guaranteed at school, leading to a negative sense of disillusionment about the future. Consequently, they may become less willing to consider and care about the feelings of others (Luengo Kanacri et al., 2017; Fite et al., 2019). On the other hand, children who experience high levels of peer victimization might acquire a lower level of theory of mind and might be aggressive, harm others, and even hurt themselves (van Geel et al., 2015). In fact, the important prerequisite for prosocial behavior is self-compassion, as indicated by Neff and Pommier (2013): loving oneself enables one to care for others. In other words, individuals can strengthen their social connections and increase prosocial behavior only when they have a foundation of self-care and self-appreciation.

### 4.2 Mediating effects of self-concept clarity and cognitive reappraisal

Consistent with our expectations, the results showed that school bullying victimization influenced social mindfulness via three pathways: SCC, cognitive reappraisal, and the chain mediating effect of SCC and cognitive appraisal; the results enable a deeper comprehension of the mechanism underlying the association between school bullying victimization and social mindfulness.

First, the partial mediation effect of SCC on the association between bullying victimization and social mindfulness was supported. Maladaptive patterns formed during childhood or adolescence can persist and manifest throughout an individual’s life, as per the schema theory, potentially leading to significant disruptions in their self-cognitive functioning. These maladaptive patterns can impact various aspects of their life, including their relationships, behaviors, and overall well-being (Young et al., 2003). The children who are not accepted by their peers might have a lower level of SCC due to conflicts and the situations they are faced with (Du et al., 2023). Peer victimization also predicts lower self-esteem, which in return leads to a higher possibility of being bullied (van Geel et al., 2018). However, having a clear self-concept has consistently been regarded as a positive self-regulatory resource (Ritchie et al., 2011). Individuals with a clear self-concept tend to maintain positive and stable relationships with others. They are also more adaptable in the face of life stressors and can effectively navigate changes in the external environment. Consequently, they tend to experience a high sense of meaning in life. A clear self-concept serves as a foundation for self-awareness, self-esteem, and personal growth, contributing to overall psychological well-being. Helping young students recognize and address these maladaptive patterns through therapy or interventions is crucial for enhancing self-perception and overall functioning.

Second, the mediation role of cognition reappraisal in the association between bullying victimization and social mindfulness was supported. As the schema theory indicates, adolescents who have experienced bullying may exhibit negative cognitive biases (Young et al., 2003). During this time, they may also use fewer cognitive reappraisal strategies to reevaluate their environments and others. These factors can impact their interaction with society and others. They may be more cautious and guarded or find it difficult to trust others due to their negative experiences (Zhang et al., 2005). Previous studies suggested that the support from parents, teachers, and friends helps individuals learn how to manage stress and adversity, improving their ability to adapt their emotions (Li et al., 2021). Another research also states that peer supports promote adolescents’ emotional support skills and mental health (Pavarini et al., 2023). Therefore, employing the cognition reappraisal strategy assists those experiencing negative emotions enhance their capacity to perceive the needs and distress of themselves and other persons, which indeed improves their social mindfulness (Hein et al., 2018). It is important to provide support and interventions to help adolescents develop healthier cognitive strategies and improve their social interactions and relationships.

Last, the chain mediation effect of SCC and cognition reappraisal was observed in the relationship between school bullying victimization and social mindfulness. As mentioned above, a key developmental task is the establishment of self-identity (Erikson, 1968). Adolescents engaging in self-exploration may experiment with different identities and engage in activities that help them define their unique sense of self. A clear self-concept during adolescence promotes self-regulation skills, reduces the inclination toward rumination, and encourages adolescents to consider situations from others’ perspectives and solve problems through positive methods such as cognition reappraisal to change their attitude toward unfriendly situation (Kural and Kovács, 2022). Additionally, Xu (2007) suggested that SCC is a buffer for stressful life events. Individuals with low SCC face challenges in extracting self-relevant information in stressful situations, leading them to rely more on external stimuli for decision-making and making them susceptible to environmental influences. This exacerbates their tendency toward self-disruption, resulting in coping strategies such as denial, psychological and behavioral disintegration, and reliance on alcohol or drugs to deal with problems.

## 5 Contributions and limitations

### 5.1 Contributions

This study examined the effects of school bullying victimization experience on social mindfulness in Chinese middle school students in light of the interdependence and schema theories. The results not only demonstrate that bullying indeed has a significantly negative influence on social mindfulness, but also shows that SCC and cognition reappraisal play protective roles against the effect of school bullying victimization on the development of social mindfulness among middle school students. In a society that highlights kindness and honesty, emphasizes group attachment and interpersonal harmony, and encourages creativity and openness, peer bullying and rejection are extremely despised while social mindfulness is highly recommended. As social mindfulness in China fits in with the concepts of respect, empathy, and virtue education, this study provides insights for reducing the negative effects of bullying experience and suggestions for improving social mindfulness among adolescents. Parents are expected to help adolescents explore themselves, figuring out who they are and their real interests and hobbies, and accompany them in viewing the world from different perspectives. Teachers can design a series of classes and situational games to assist students in building their self-identity, develop options to deal with stressful events, and adopt strategies to efficiently cope with negative experiences. Mental health workers need to address self-cognitive and coping strategies when working with young adolescents with a history of bullying.

### 5.2 Limitations

There are some limitations to this study: (1) Only the questionnaire method was used in this cross-sectional study, which could not fully clarify the causal relationships among variables. Subsequent studies could employ experimental methods or conduct longitudinal investigations to further clarify the relationship between variables. (2) Regarding the mechanism underlying the effect of bullying victimization on social mindfulness, we only focused on SCC and cognition reappraisal. In fact, other factors such as empathy and moral cognition also affect the prosocial attitudes of bullying victims. Future studies can expand this field by exploring different mechanisms to help the victims build altruistic cognition while preventing them from becoming bullies.

## Data availability statement

The raw data supporting the conclusions of this article will be made available by the authors, without undue reservation.

## Ethics statement

The studies involving humans were approved by the Ethic Committee of the College of Psychology of Sichuan Normal University. The studies were conducted in accordance with the local legislation and institutional requirements. Written informed consent for participation in this study was provided by the participants' legal guardians/next of kin.

## Author contributions

WY: Conceptualization, Investigation, Methodology, Writing – original draft. DH: Data curation, Software, Validation, Writing – original draft. YG: Project administration, Supervision, Visualization, Writing – review & editing.
